# Antimicrobial Susceptibility of Community-Acquired Urine Bacterial Isolates in French Amazonia

**DOI:** 10.4269/ajtmh.22-0242

**Published:** 2023-04-03

**Authors:** Flaubert NkontCho, Vincent Sainte-Rose, Philippe Abboud, Patrick Portecop, Jean Marc Pujo, Fabrice Cook, Gaelle Walter, Roman Mounier, Dabor Resiere, Stephanie Houcke, Magalie Demar, Hatem Kallel, Felix Djossou

**Affiliations:** 1 Pharmacy Department, Cayenne General Hospital, Cayenne, French Guiana;; 2 Laboratory of Microbiology, Cayenne General Hospital, Cayenne, French Guiana;; 3 Tropical and Infectious Diseases Department, Cayenne General Hospital, Cayenne, French Guiana;; 4 Emergency Department, Guadeloupe University Hospital, Pointe-à-Pitre, Guadeloupe;; 5 Emergency Department, Cayenne General Hospital, Cayenne, French Guiana;; 6 Intensive Care Unit, Cayenne General Hospital, Cayenne, French Guiana;; 7 Neuro-Intensive Care Unit, GHU-Paris, Paris University, Paris, France;; 8 Intensive Care Unit, Martinique University Hospital, Fort de France, Martinique;; 9 Tropical Biome and Immunopathology CNRS UMR-9017, INSERM U 1019, Université de Guyane, Cayenne, French Guiana

## Abstract

Bacterial resistance in community-acquired urinary tract infections (UTIs) is increasing worldwide. Our study aimed to assess the microbiological epidemiology and antimicrobial susceptibility patterns of community-acquired urine bacterial isolates in French Amazonia. Our study is retrospective. It was conducted from January 2015 to December 2019 in the microbiology laboratory of the Cayenne General Hospital (French Guiana). It includes all positive urine samples from adult (> 18 years) outpatients (*N* = 2,533). Isolated microorganisms were Gram-negative rods in 83.9%, mainly *Enterobacterales* (98.4%). The main isolated bacteria were *Escherichia coli* (58.7%) and *Klebsiella pneumoniae* (13.3%). Among the isolated *E. coli*, 37.2% were susceptible to amoxicillin, 77.9% to amoxicillin/clavulanic acid, 94.9% to cefotaxime, 78.9% to ofloxacin, and 98.9% to nitrofurantoin. In 106 cases (5.1%), isolated *Enterobacterales* were extended-spectrum β-lactamase producers (5% of *E. coli* and 8.9% of *K. pneumoniae*). Overall, high levels of cross- and co-resistance were registered. The main isolated Gram-positive bacteria was *Staphylococcus saprophyticus* (28.9%). It was resistant to oxacillin in 52.5% of cases and susceptible to nitrofurantoin in 99.1% of cases. Patients with *S. saprophyticus* were young women in almost all cases. In conclusion, the most isolated microorganisms from outpatient urinalyses were *E. coli* and *K. pneumoniae*. They showed a high resistance rate to amoxicillin, but they were susceptible to the most remaining antibiotics. *S. saprophyticus* was isolated mainly in young women and was resistant to oxacillin in half of the cases. Interestingly, nitrofurantoin was active against most isolated organisms and can be considered as empirical treatment in uncomplicated UTIs.

## INTRODUCTION

Antibiotic resistance is increasing worldwide, resulting in a serious threat problem in the community.^1^ In Latin America, resistance in community-acquired urinary tract infections (UTIs) is also increasing.^2^ In French Guiana, Baizet et al.,^3^ reported in a retrospective study of adult patients diagnosed with community-acquired UTI that *Escherichia coli* was predominant (74.1%) and had decreased susceptibility to ampicillin, amoxicillin/clavulanic acid, fluoroquinolones, cotrimoxazole, and furans compared with the susceptibility profile observed in mainland France. In that study, 3.1% of *E. coli* and 31.6% of *Klebsiella pneumoniae* were extended-spectrum β-lactamase (ESBL) producers.^3^

Urinary tract infection is a common infectious presentation in community practice worldwide. It is often treated with broad-spectrum antibiotics because of concerns about infection with resistant organisms. Consequently, the extensive use of antimicrobial agents has invariably resulted in the development of antibiotic resistance. Indeed, antibiotic resistance in uropathogens has changed in recent years, in both the community and hospitals.^4^^,^^5^ In addition, there is little available information on the resistance pattern of microorganisms causing community-acquired UTIs in French Amazonia.

Initial antibiotic treatment of UTIs is typically empirical, and the appropriate treatment is initiated after urine culture and susceptibility tests. The empiric treatment should include an antimicrobial to which all probable uropathogens are susceptible.^6^ For this, it is essential to know the most common etiological agents for UTIs, and antibiotic resistance rates in the related geographic area because resistance rates in different geographic regions can vary.^7^ Additionally, most antibiotics are eliminated by glomerular filtration and high antibiotics concentrations were documented in the urines of treated patients.^8^ This raises the question of whether antibiotic resistance is a major concern in treating UTIs. Indeed, resistance or susceptible profile are defined in vitro according to the minimal inhibitory concentration of the antibiotic on the causal bacteria. High levels of antibiotics in urines and the kidney parenchyma suggest that some antibiotics can effectively treat UTIs despite their in vitro resistance profile. For this, some authors encourage rethinking urinary antibiotic breakpoints.^8^ In addition to the resistance levels,^9^ monitoring co- and cross-resistance to the available antibiotics is important to select appropriate alternatives.

We conducted this retrospective study to search for the microbiological epidemiology and antimicrobial susceptibility patterns of community-acquired urine bacterial isolates in French Amazonia.

## MATERIALS AND METHODS

We conducted this retrospective study over 5 years (January 2015 to December 2019) in the microbiology laboratory of the Cayenne General Hospital (French Guiana). We included all community-acquired isolates in urine samples from adult patients (> 18 years) attending the emergency department (ED), outpatient clinics (OC), or remote health care centers (RHCC) with a significant density of growth independently of the clinical diagnosis of UTI and the prior antibiotics exposure. The Cayenne general hospital is a 742-bed general center that provides first-line medical care for an urban population of 150,000. It manages 18 RHCC, providing care for additional 50,000 inhabitants. It is also a referral center for a larger population coming from all over French Guiana and the border countries.^10^

We reviewed all positive urine cultures and antibiotic susceptibility testing. Fungal cultures, anaerobic bacterial cultures, polymicrobial cultures, and negative cultures were excluded from this study.

### Data collection.

We collected data from the computerized database of the microbiology laboratory. A community-acquired isolate is defined as a culture collection from an outpatient (i.e., consulting in the ED, OC, or RHCC). Significant density of growth refers to the breakpoints defined by the French society of microbiology.^11^ In women, they are defined as ≥ 10^3^ colony-forming units (CFU)/mL for *E. coli* and *Staphylococcus saprophiticus*; ≥ 10^4^ CFU/mL for enterobacterales and *Pseudomonas aeruginosa*; and ≥ 10^5^ CFU/mL for *Streptococcus agalactiae*, nonsaprophiticus coagulase-negative staphylococci (CNS), and non–*P. aeruginosa* nonfermentative Gram-negative bacteria (GNB). In men, they are defined as ≥ 10^3^ CFU/mL for *E. coli, S. saprophiticus*, *Enterobacterales*, and *P. aeruginosa*; and ≥ 10^5^ CFU/mL for *S. agalactiae*, nonsaprophiticus CNS, and non–*P. aeruginosa* non fermentative GNB. Redundant cases are defined as positive culture urinalysis in the same patient growing to the same organism in an interval of < 6 months. Each patient with nonredundant results is considered as a new patient. *Enterobacterales* were divided into three groups according to their enzymatic resistance profile to β-lactams at the basal state (natural resistance).^12^ Group I are those without enzymatic resistance at the basal state (e.g., *E. coli*, *Proteus mirabilis*), group II are those producing low-level penicillinase (e.g., *K. pneumoniae*, *Citrobacter koseri*), and group III are those producing low-level AmpC (e.g., *Enterobacter *spp.,* Serratia marcessens, Citrobacter freundii*). Cross-resistance refers to resistance to several antibiotics with a similar mechanism of action. Coresistance refers to resistance to more than one class of antibiotics. For each included urine culture, we collected the patient’s age and gender, the isolated bacteria, and its susceptibility to different classes of antibiotics.

### Microbiological technique.

Bacterial inoculation was performed on Uriselect^®^ media (Bio-Rad, Marnes-la-Coquette, France) incubated at 35–37°C for 18 to 24 hours under an aerobic atmosphere. Indole positive pink colonies were identified as *E. coli*; other bacteria were identified by mass spectrometry (MALDI biotyper—Bruker). The antimicrobial susceptibility tests were performed by using VITEK 2 AST-N372 card (BioMérieux, Marcy l’Étoile, France) or by disk diffusion method in Mueller–Hinton medium (BioMérieux). The susceptibility to antibiotics was estimated according to the Antibiogram Committee of the French Society for Microbiology.^13^ The identification of ESBL-producing enterobacterales (ESBL-PE) was confirmed by the disc diffusion method to detect synergy.^14^

### Statistical analysis.

Results are reported as median and interquartiles ranges (IQR: 1st–3rd quartiles) or numbers with percentages. Qualitative variables were compared using Fisher’s exact test, and continuous variables the Mann–Whitney *U* test. We used linear regression, and calculated the correlation coefficient (*R*^2^) to determine the trend of the event’s prevalence (susceptibility to the studied antibiotic) according to the quarter of the study. Statistical significance was defined as a *P* value ≤ 0.05. Statistical analyses were carried out with Excel (2010 Microsoft Corporation, Redmond, WA) and IBM SPSS Statistics for Windows, version 24 (IBM Corp., Armonk, NY).

### Ethical considerations.

This study was approved by the ethics committee of our hospital. Written information was distributed to all patients or their relatives, stating that their data could be used for research purposes and that they can oppose that. Our database has been registered at the Commission National de l’Informatique et des Libertés (registration no. 2217629), complying with French law on electronic data sources.

## RESULTS

**Figure 1.**
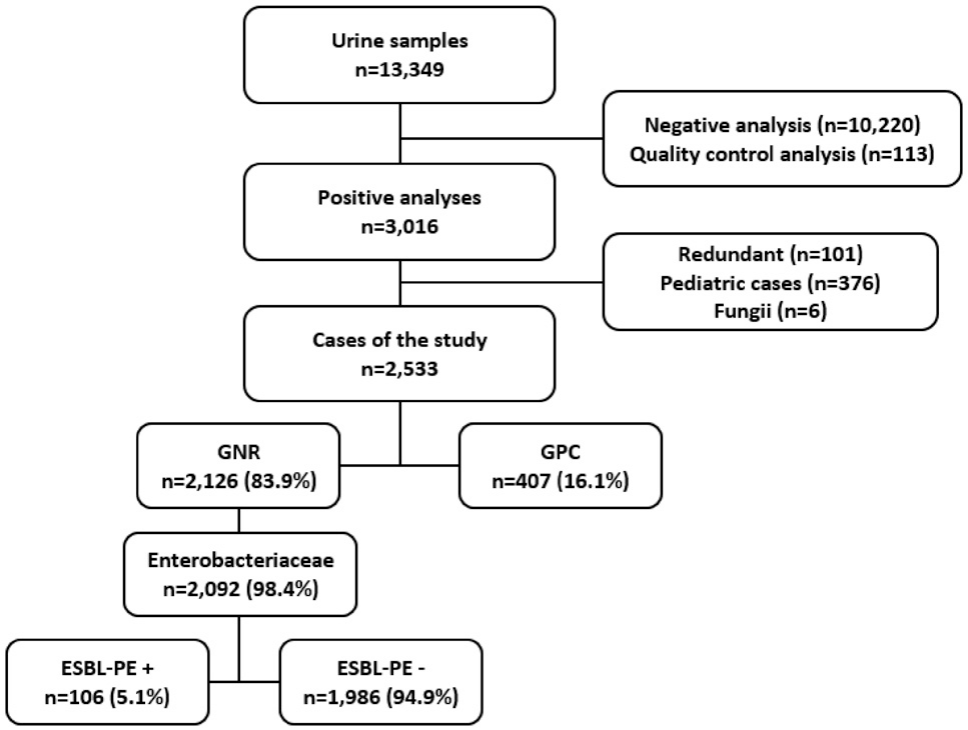
Flow chart of the study. ESBL-PE = extended-spectrum β-lactamase producer Enterobacterales; GNR = Gram-negative rods; GPC = Gram-positive cocci.

During the study period, 13,349 urinalyses were performed in our bacteriology laboratory. After exclusions, 2,533 positive urinalyses were studied (Figure 1). The distribution of the exams according to the year of the study showed an average of 635 positive urinalyses per year. The urinalyses were sampled in the emergency department in 64% of cases, in the remote healthcare centers in 29% of cases, and in the outpatient clinics in 7% of cases. Patients were women in 74.1% of cases. The median age of patients was 44 years (IQR: 30–66). It was 38 (IQR: 28–57) in women and 63 (IQR: 46–76) in men (*P* < 0.001).

**Figure 2.**
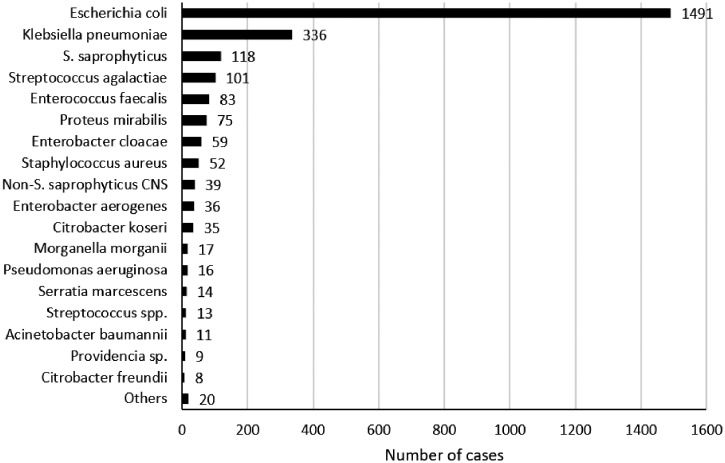
The microorganisms isolated in urine cultures. CNS = coagulase-negative staphylococci.

The distribution of the isolated microorganisms is reported in Figure 2. Isolated microorganisms were Gram-negative rods (GNR) in 2,126 cases (83.9%) and Gram-positive cocci (GPC) in 407 cases (16.1%). Among the isolated GNR, 2,092 (98.4%) were *Enterobacterales*. They were group I *Enterobacterales* in 1,572 cases (75%), group II in 374 cases (17.9%), and group III in 146 cases (7.1%) (Table 1). The main isolated bacteria were *E. coli* (1,491 cases; 58.7%) and *K. pneumoniae* (336 cases; 13.3%). Patients with *E. coli* were 42 years old (IQR: 30–63), and 79.1% were women. Patients with *K. pneumoniae* were 52 years old (IQR: 31–72), and 68.8% of them were women (*P* < 0.001 for both values compared with *E. coli* group).

**Table 1:** Susceptibility profile of Gram-negative rods recovered from urine samples

Tested antibiotics	GNR		*Enterobacterales*		Group I EB		Group II EB		Group III EB		NF-GNR	
	Nb	Result	Nb	Result	Nb	Result	Nb	Result	Nb	Result	Nb	Result
ESBL-PE	2,092	106 (5.1%)	2,092	106 (5.1%)	1,572	75 (4.8%)	374	30 (8%)	146	1 (0.7%)	29	0 (0%)
Penicillins												
Ampicillin	2,100	608 (29%)	2,092	608 (29.1%)	1,572	608 (38.7%)	374	0 (0%)	146	0 (0%)	–	–
Amoxicillin/clavulanic acid	2,099	1,566 (74.6%)	2,092	1,565 (74.8%)	1,572	1,240 (78.9%)	374	325 (86.9%)	146	0 (0%)	–	–
Piperacillin	2,124	732 (34.5%)	2,092	706 (33.7%)	1,572	610 (38.8%)	374	0 (0%)	146	96 (65.8%)	29	26 (89.7%)
Ticarcillin	2,125	752 (35.4%)	2,092	725 (34.7%)	1,572	629 (40%)	374	0 (0%)	146	96 (65.8%)	29	27 (93.1%)
Piperacillin/tazobactam	2,124	2,012 (94.7%)	2,091	1,982 (94.8%)	1,571	1,502 (95.6%)	374	344 (92%)	146	136 (93.2%)	29	28 (96.6%)
Temocillin	1,318	1,286 (97.6%)	1,317	1,286 (97.6%)	1,044	1,030 (98.7%)	193	186 (96.4%)	80	70 (87.5%)	–	–
Mécillinam	1,055	970 (91.9%)	1,055	970 (91.9%)	1,004	933 (92.9%)	44	34 (77.3%)	7	3 (42.9%)	–	–
Aztreonam	687	622 (90.5%)	669	611 (91.3%)	625	585 (93.6%)	37	21 (56.8%)	7	5 (71.4%)	16	11 (68.8%)
Cephalosporins												
Cefotaxime	2,096	1,971 (94%)	2,092	1,968 (94.1%)	1,572	1,495 (95.1%)	374	344 (92%)	146	129 (88.4%)	–	–
Ceftazidime	2,126	2,011 (94.6%)	2,092	1,982 (94.7%)	1,572	1,507 (95.9%)	374	344 (92%)	146	131 (89.7%)	29	26 (89.7%)
Cefepime	2,126	2,017 (94.9%)	2,092	1,984 (94.8%)	1,572	1,507 (95.9%)	374	346 (92.5%)	146	131 (89.7%)	29	29 (100%)
Carbapenems												
Imipenem	2,124	2,118 (99.7%)	2,091	2,086 (99.8%)	1,572	1,571 (99.9%)	374	374 (100%)	145	141 (97.2%)	29	29 (100%)
Meropenem	121	120 (99.2%)	90	90 (100%)	48	48 (100%)	35	35 (100%)	7	7 (100%)	29	29 (100%)
Ertapenem	2,080	2,076 (99.8%)	2,078	2,074 (99.8%)	1,567	1,567 (100%)	368	367 (99.7%)	143	140 (97.9%)	–	–
Aminoglycosides												
Amikacin	2,120	2,084 (98.3%)	2,089	2,054 (98.3%)	1,570	1,546 (98.5%)	373	364 (97.6%)	146	144 (98.6%)	29	28 (96.6%)
Tobramycin	1,185	1,080 (91.1%)	1,154	1,049 (90.9%)	879	805 (91.6%)	203	183 (90.1%)	72	61 (84.7%)	29	29 (100%)
Gentamycin	2,111	1,960 (92.8%)	2,080	1,929 (92.7%)	1,564	1,453 (92.9%)	373	351 (94.1%)	143	125 (87.4%)	29	29 (100%)
Fluoroquinolones												
Ofloxacin	2,097	1,710 (81.5%)	2,092	1,707 (81.6%)	1,572	1,256 (79.9%)	374	328 (87.7%)	146	123 (84.2%)	–	–
Ciprofloxacin	1,977	1,797 (90.9%)	1,943	1,766 (90.9%)	1,449	1,311 (90.5%)	362	331 (91.4%)	132	124 (93.9%)	29	28 (96.6%)
Others												
TMP/SMX	2,097	1,402 (66.9%)	2,082	1,387 (66.6%)	1,567	955 (60.9%)	372	317 (85.2%)	143	115 (80.4%)	13	13 (100%)
Nitrofurantoin	2,084	1,786 (85.7%)	2,083	1,785 (85.7%)	1,565	1,475 (94.2%)	373	238 (63.8%)	145	72 (49.7%)	–	–
Fosfomycin	765	750 (98%)	764	749 (98%)	714	708 (99.2%)	43	35 (81.4%)	7	6 (85.7%)	–	–

EB = Enterobacteriaceae; ESBL = extended-spectrum β-lactamases; GNR = Gram-negative rods; Nb = number of tested isolates; NF-GNR = nonfermentative GNR; PE = producing enterobacterales; TMP/SMX = trimethoprim/sulfamethoxazole.

The susceptibility of GNR to antimicrobials is reported in Table 1. Antibiotic susceptibilities of *E. coli* and *K. pneumoniae* are reported in Table 2. Among the 1,491 tested *E. coli*, 37.2% were susceptible to amoxicillin, 77.9% to amoxicillin/clavulanic acid, 94.9% to cefotaxime, 100% to imipenem, 98.5% to amikacin, 78.9% to ofloxacin, and 98.9% to nitrofurantoin.

**Table 2:** Susceptibility profile of isolated *Escherichia coli* and *Klebsiella pneumoniae*

Tested antibiotics	*E. coli* and *K. pneumoniae*		*E. coli*		*K. pneumoniae*	
	Nb	Result	Nb	Result	Nb	Result
ESBL	1,827	104 (5.7%)	1,491	74 (5%)	336	30 (8.9%)
Penicillins						
Amoxicillin	1,827	554 (30.3%)	1,491	554 (37.2%)	336	0 (0%)
Amoxicillin/clavulanic acid	1,827	1,450 (79.4%)	1,491	1,162 (77.9%)	336	288 (85.7%)
Piperacillin	1,827	555 (30.4%)	1,491	555 (37.2%)	336	0 (0%)
Piperacillin/Tazobactam	1,826	1,727 (94.6%)	1,490	1,421 (95.4%)	336	306 (91.1%)
Temocillin	1,138	1,117 (98.2%)	964	950 (98.5%)	174	167 (96%)
Mecillinam	1,042	961 (92.2%)	1,001	930 (92.9%)	41	31 (75.6%)
Cephalosporins						
Cefotaxime	1,827	1,721 (94.2%)	1,491	1,415 (94.9%)	336	306 (91.1%)
Ceftazidime	1,827	1,733 (94.9%)	1,491	1,427 (95.7%)	336	306 (91.1%)
Cefepime	1,827	1,735 (95%)	1,491	1,427 (95.7%)	336	308 (91.7%)
Carbapenems						
Imipenem	1,827	1,827 (100%)	1,491	1,491 (100%)	336	336 (100%)
Ertapenem	1,817	1,816 (99.9%)	1,487	1,487 (100%)	330	329 (99.7%)
Aminoglycosides						
Amikacin	1,824	1,792 (98.2%)	1,489	1,466 (98.5%)	335	326 (97.3%)
Tobramycin	1,023	930 (90.9%)	839	766 (91.3%)	184	164 (89.1%)
Gentamycin	1,819	1,687 (92.7%)	1,483	1,373 (92.6%)	336	314 (93.5%)
Fluoroquinolones						
Ofloxacin	1,827	1,467 (80.3%)	1,491	1,177 (78.9%)	336	290 (86.3%)
Ciprofloxacin	1,692	1,525 (90.1%)	1,368	1,232 (90.1%)	324	293 (90.4%)
Others						
TMP/SMX	1,820	1,166 (64.1%)	1,486	886 (59.6%)	334	280 (83.8%)
Nitrofurantoin	1,819	1,671 (91.9%)	1,484	1,468 (98.9%)	335	203 (60.6%)
Fosfomycin	751	737 (98.1%)	711	705 (99.2%)	40	32 (80%)

Nb = number of tested isolates; TMP/SMX = trimethoprim/sulfamethoxazole.

**Figure 3.**
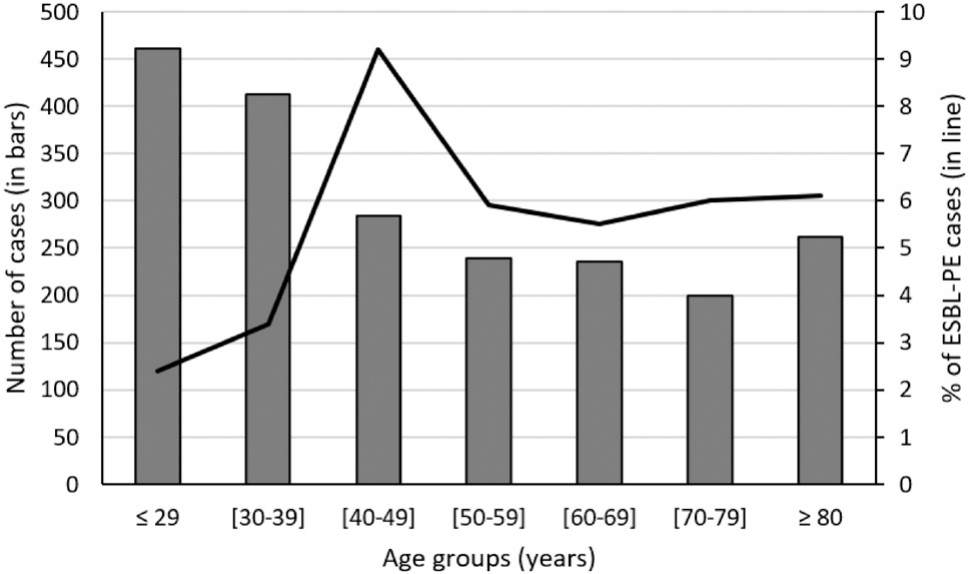
Percentage of extended spectrum β-lactamase producing Enterobacteriaceae (ESBL-PE) (in line) according to the age group (in bars).

**Figure 4.**
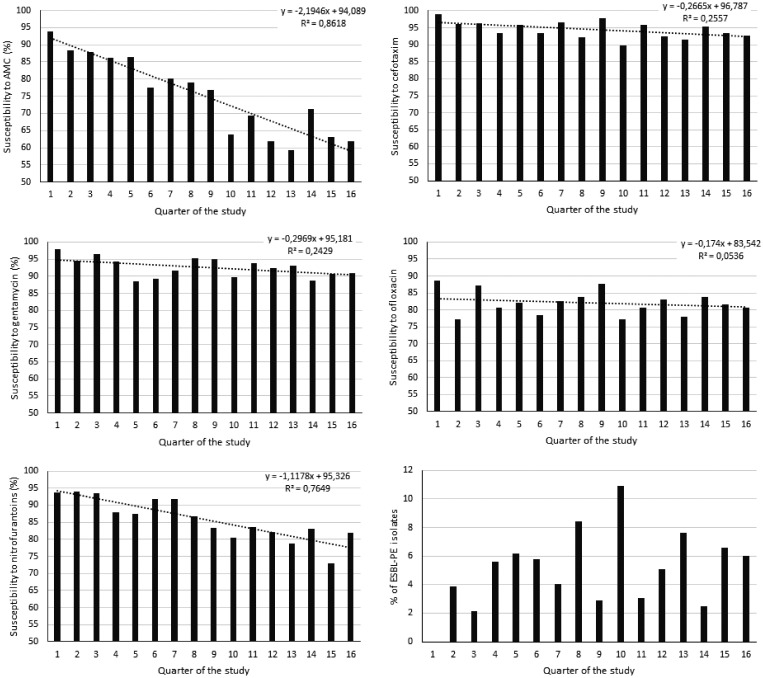
The susceptibility trend of *Enterobacterales* and extended-spectrum β-lactamase producing Enterobacteriaceae (ESBL-PE) profile across the 16 quarters of the study. *P* value was < 0.001 for amoxicillin/clavulanate, 0.045 for cefotaxime, 0.052 for gentamycin, 0.388 for ofloxacin, and < 0.001 for nitrofurantoin. AMC = amoxicillin/clavulanic acid.

In 106 cases (5.1%), isolated *Enterobacterales* were ESBL producers (5% of *E. coli* and 8.9% of *K. pneumoniae*; *P* = 0.008). The patient’s age was 52 years (IQR: 40–70) in patients with ESBL-PE versus 45 (31–67) in those without (*P* = 0.002). The female gender was 60% in patients with ESBL-PE versus 74.9% in those without (*P* = 0.001). The percentages of ESBL-PE according to the age groups are reported in Figure 3. ESBL-PE rate was 4.8% in group I, 8% in group II, and 0.7% in group III *Enterobacterales*. Most of the isolated ESBL-PE in urinalyses were sampled in the ED and the outpatient clinics. The susceptibility trend of *Enterobacterales* and ESBL-PE profile across the 16 quarters of the study showed a decreasing susceptibility profile for amoxicillin/clavulanic acid (*P* < 0.001), cefotaxime (*P* = 0.045), and nitrofurantoin (*P* < 0.001). Although the susceptibility trends for gentamycin (*P* = 0.052) and ofloxacin (*P* = 0.388) were stable (Figure 4). Cross- and co-resistance among *Enterobacterales* are reported in Table 3.

**Table 3:** Cross-resistance and coresistance among Enterobacterales isolated from urine samples

Tested antibiotics	AMX	AMC	PTZ	TEM	CTX	CAZ	IPM	AMK	GEN	OFL	CIP	SXT	FT	FS
ESBL-PE	100	78.3	38.7	22.8	100	100	0.0	24.5	44.3	88.7	83.8	77.9	16.8	12.2
AMX	100	35.5	7.3	4.4	8.4	7.4	0.3	2.2	10.1	23.0	12.0	44.8	16.7	2.8
AMC	100	100	20.5	6.7	19.2	16.5	0.8	5.1	17.5	30.6	16.0	51.1	20.2	3.7
PTZ	100	99.1	100	22.9	41.3	38.5	0.9	12.0	29.9	51.4	40.2	61.5	19.8	9.0
TEM	100	77.4	51.6	100	51.6	48.4	3.2	9.7	53.3	54.8	44.0	48.1	36.7	10.0
CTX	100	81.5	36.3	23.5	100	88.7	1.6	21.8	46.0	84.7	78.3	75.2	23.5	12.0
CAZ	100	79.1	38.2	24.6	100	100	1.8	21.8	50.0	83.6	78.0	73.1	23.8	13.8
IPM	100	80.0	20.0	20.0	40.0	40.0	100	0.0	0.0	20.0	20.0	20.0	100	0.0
AMK	94.3	77.1	37.1	15.0	77.1	68.6	0.0	100	57.1	91.4	86.4	77.1	29.4	12.0
GEN	98.7	60.3	21.2	18.6	37.7	36.4	0.0	13.2	100	75.5	57.3	75.5	18.4	9.5
OFL	88.8	41.8	14.5	8.2	27.3	23.9	0.3	8.3	29.6	100	75.0	65.5	12.9	4.7
CIP	91.5	40.1	20.9	22.9	40.7	36.2	0.6	10.7	33.3	100	100	67.4	13.4	10.3
TMP/SMX	95.1	38.1	9.2	3.7	13.1	11.4	0.1	3.9	16.0	36.1	19.7	100	8.4	3.4
FT	82.6	35.2	7.0	4.8	9.4	8.4	1.7	3.4	9.1	16.4	8.4	19.5	100	11.5
FS	93.3	73.3	40.0	20.0	73.3	73.3	0.0	20.0	57.1	60.0	53.8	78.6	21.4	100

AMC = amoxicillin/clavulanic acid; AMK = amikacin; AMX = amoxicillin; CAZ = ceftazidime; CIP = ciprofloxacin; CTX = cefotaxime; ESBL-PE = extended-spectrum β-lactamase producing Enterobacteriaceae; FS = fosfomycin; FT = nitrofurantoin; GEN = gentamycin; IPM = imipenem; PTZ = piperacillin/tazobactam; OFL = ofloxacin, TMP/SMX = trimethoprim/sulfamethoxazole; TEM = temocillin. Values indicate the resistance rate to the antibiotic in the column when the strain is resistant to the antibiotic in the row.

GPCs were isolated in 407 cases (16.1%). They were CNS in 157 cases (38.6%), *S. agalactiae* in 101 cases (24.8%), *Enterococcus faecalis* in 83 cases (20.4%), and *Staphylococcus aureus* in 52 cases (12.8%). CNS was *S. saprophyticus* in 118 cases (75.2% of CNS). It was resistant to oxacillin in 52.5% of cases and susceptible to nitrofurantoin and tetracycline in 99 and 90% of cases respectively. Patients with *S. saprophyticus* were women in 94.5% of cases and were 29 years old (IQR: 21–36). *S. aureus* was resistant to oxacillin in 13.5% of cases. Susceptibility profiles of GPC are reported in Table 4.

**Table 4:** Susceptibility profile of Gram-positive cocci isolates from urine samples

Tested antibiotics	GPC		CNS		*S. saprophyticus*		*S. aureus*		*S. agalactia*		*E. faecalis*	
	Nb	Result	Nb	Result	Nb	Result	Nb	Result	Nb	Result	Nb	Result
Penicillins												
Penicillin G	213	36 (16.9%)	157	23 (14.6%)	118	16 (13.6%)	52	9 (17.3%)	4	4 (100%)	0	–
Oxacillin	209	113 (54.1%)	157	68 (43.3%)	118	56 (47.5%)	52	45 (86.5%)	0	–	0	–
Amoxicillin	185	184 (99.5%)	–	–	–	–	–	–	101	101 (100%)	83	83 (100%)
Aminoglycosides												
Kanamycin	209	197 (94.3%)	157	146 (93%)	118	115 (97.5%)	52	51 (98.1%)	–	–	–	–
Tobramycin	209	197 (94.3%)	157	146 (93%)	118	115 (97.5%)	52	51 (98.1%)	–	–	–	–
Gentamycin	209	198 (94.7%)	157	146 (93%)	118	115 (97.5%)	52	52 (100%)	–	–	–	–
Macrolids												
Tetracyclin	218	166 (76.1%)	157	122 (77.7%)	118	106 (89.8%)	52	43 (82.7%)	9	1 (11.1%)	–	–
Erythromycin	267	123 (46.1%)	157	77 (49%)	118	55 (46.6%)	52	38 (73.1%)	9	7 (77.8%)	49	1 (2%)
Lincomycin	203	189 (93.1%)	153	139 (90.8%)	114	104 (91.2%)	50	50 (100%)	–	–	–	–
Fluoroquinolones												
Ofloxacin	209	183 (87.6%)	157	139 (88.5%)	118	111 (94.1%)	52	44 (84.6%)	–	–	–	–
Glycopeptides												
Vancomycin	268	265 (98.9%)	157	156 (99.4%)	118	117 (99.2%)	52	52 (100%)	9	9 (100%)	49	48 (98%)
Others												
TMP/SMX	256	189 (73.8%)	157	107 (68.2%)	118	79 (66.9%)	52	48 (92.3%)	–	–	47	34 (72.3%)
Rifampicin	210	201 (95.7%)	157	149 (94.9%)	118	115 (97.5%)	52	52 (100%)	–	–	–	–
Nitrofurantoin	253	251 (99.2%)	146	145 (99.3%)	109	108 (99.1%)	49	49 (100%)	9	8 (88.9%)	49	49 (100%)
Fosfomycin	209	57 (27.3%)	157	6 (3.8%)	–	–	52	51 (98.1%)	–	–	–	–
Fucidic acid	209	68 (32.5%)	157	22 (14%)	–	–	52	46 (88.5%)	–	–	–	–
Linezolid	259	254 (98.1%)	149	144 (96.6%)	112	107 (95.5%)	51	51 (100%)	9	9 (100%)	49	49 (100%)

CNS = coagulase-negative staphylococci; GPC = Gram-positive cocci; Nb = number of tested isolates; TMP/SMX = trimethoprim/sulfamethoxazole.

## DISCUSSION

Our study shows that the most isolated microorganisms from outpatient urinalyses were *Enterobacterales*, mainly *E. coli* and *K. pneumoniae*. They were susceptible to most antibiotics (wild type) in most cases. We highlight an elevated resistance level of *Enterobacterales* to amoxicillin and a high susceptibility rate to nitrofurantoin and fosfomycin. Additionally, we found a decreasing susceptibility profile in *Enterobacterales* for amoxicillin/clavulanate, cefotaxime, and nitrofurantoin across the 16 quarters of the study.

In Amazonia, Baizet et al.^3^ conducted a retrospective study of adults attending the ED of Cayenne Hospital with a diagnosis of UTI. They found that *E. coli* was predominant (74.1%). We indeed found 5 years later that *Enterobacterales* mainly *E. coli*, as the first isolate from urine cultures (58.7% in absolute). Despite a high representation of GPCs (16.1%), the flora and the resistance profile found in our study are different from those reported in Latin America.^2^ This is probably explained by the flow of populations in French Guiana, mostly from the French islands and the European continent rather than from the surrounding countries.

*Enterobacterales* were resistant to amoxicillin in 70.9%, to amoxicillin/clavulanic acid in 25.2%, and to cefotaxime in 5.9% of cases. Interestingly, they were sensitive to fosfomycin and nitrofurantoin in 98% and 86% of cases, respectively. For this, fosfomycin and nitrofurantoin seem to be a reasonable option for empirical treatment of uncomplicated UTIs.^15^^–^^18^ Indeed, fosfomycin and nitrofurantoin are active against common causes of UTIs, mainly *E. coli*, whereas nonfermentative GNRs are naturally resistant. Overall, resistance to these two antibiotics is uncommon in *Enterobacterales* and many multidrug resistant organisms retain susceptibility.^15^ On the other hand, fluoroquinolones were active against 90% of *Enterobacteriaceae* isolates. They were reported as effective for clinical and microbiological cures in patients with uncomplicated UTIs.^19^ However, they should be spared in the first- and second-line UTI treatment because of their selection pressure and also because they should be saved for more severe infections.^19^^,^^20^ On the other hand, our study shows that the susceptibility trend of *Enterobacterales* showed a decreasing susceptibility profile over time for amoxicillin/clavulanic acid, cefotaxime, and nitrofurantoin. In contrast, the susceptibility trends for gentamycin and ofloxacin were stable. This can be explained by the antibiotic pressure in the community.

In our study, 407 specimens grew to GPC, 157 of them (38.6% of GPCs and 5.8% of all positive urinalyses) grew to CNS, and 118 to *S. saprophyticus* (75.2% of CNS). It is well known that CNS—namely, *S. saprophyticus*—can cause community-acquired UTI. Indeed, *S. saprophyticus* is part of the normal human flora that colonizes the perineum, urinary, and gastrointestinal tracts. It causes 5% to 20% of community-acquired UTIs^21^ and up to 42% of UTIs among 16- to 25-year-old women.^22^ In our study, microbiological results showed that *S. saprophyticus* was resistant to oxacillin in 52.5% of cases and was susceptible to nitrofurantoin and tetracycline in 99% and 90% of cases, respectively. Patients were young women in the majority as described in the literature.^23^ It is noteworthy that UTI symptoms caused by *S. saprophyticus* are similar but can be more severe than in patients with *E. coli* UTIs, and 40% of patients present with acute pyelonephritis.^23^

Community-acquired UTIs caused by multidrug-resistant bacteria has become a growing and challenging to treat concern^24^^,^^25^ with decreased susceptibility to ampicillin, amoxicillin/clavulanic acid, fluoroquinolones, cotrimoxazole, and furans.^9^^,^^26^ Indeed, the reported resistance rates were 21% to 63.4% for ampicillin, 1.2% to 9.6% for amoxicillin/clavulanic acid, 1% to 5.4% for cefuroxime, 0.5% to 12.9% for ciprofloxacin, 14% to 45.4% for trimethoprim-sulfamethoxazole, 0% to 2.9% for fosfomycin, and 6.3% to 32.6% for nalidixic acid.^27^ Our study shows similar results with a decreased susceptibility of *E. coli* to amoxicillin, quinolones, and to trimethoprim/sulfamethoxazole. However, the isolated *E. coli* had a higher susceptibility rate to amoxicillin/clavulanic acid and full susceptibility to furans. Regarding ESBL-PE rate, it requires close monitoring and a high-priority prevention strategy. Overall, local susceptibility rates are compatible with the antibiotics recommended by the French society of infectious diseases for the treatment of community UTI^9^^,^^26^ and therefore reinforce their relevance in our context.

ESBL-PE in the community is a significant concern worldwide. Surveillance networks revealed a predominance of ESBL-P *K. pneumoniae* in Latin America (44%) and Asia Pacific regions (22%), with a lower incidence in Europe (13.3%) and North America (7.5%).^28^^,^^29^ Moreover, *E. coli* producing ESBL type CTX-M in the community are endemic in Asia, South America, and Europe.^30^ In Latin America, the incidence rate of ESBL-PE is among the highest in the world, varying from 45% to 51% for *K. pneumoniae* and 8.5% to 18% for *E. coli*.^31^^,^^32^ In addition, ESBL production can be worsened by developing combined resistance mainly to fluoroquinolones and aminoglycosides in *E. coli* and *K. pneumoniae*. In our study, combined resistance was 88.7% to ofloxacin and 24.5% to amikacin in case of ESBL production in *Enterobacterales*. ESBL-PE among UTI agents has widely been reported and should be suspected mainly in case of prior exposure to antibiotics.^33^^–^^35^ MacVane et al.^36^ showed that the main involved ESBL-PE among UTI isolates are *E. coli* and *K. pneumoniae*. They result in an ineffective empiric antibiotic treatment (62% versus 6%, *P* < 0.001) and a delayed effective antibiotic therapy (51 versus 2.5 hours, *P* < 0.001) compared with non-ESBL organisms. In addition, they are responsible for prolonged hospital stays (6 days versus 4 days, *P* = 0.02) and higher hospital costs. They were also responsible for higher infection-related mortality (7.2% versus 1.8%) and readmission rates in 30 days (7.2% versus 3.6%) without reaching the significance level.^36^ In French Guiana, Baizet et al.^3^ reported in a retrospective study of adults consulting at the ED of Cayenne Hospital with a diagnosis of UTI, that ESBL production was detected in 3.1% of *E. coli* and 31.6% of *K. pneumoniae*. Our study diagnosed ESBL-PE in 106 cases (5.1% of isolated *Enterobacterales*). It was 5% among *E. coli* and 8.9% among *K. pneumoniae* isolates. These results are concordant with those reported by former surveys^3^^,^^37^ and approximate the figures of mainland France. Unlike its neighbors in Latin America, the French Amazonia seems to be spared from the South America continental dissemination of ESBL-PE in the community.^2^ Nevertheless, a robust prevention strategy is required to stop the spread of antibiotic resistance. Further, cross-resistance to β-lactams and coresistance to other classes of antimicrobials in ESBL-PE are frequent.^38^ Indeed, coresistance to fluoroquinolones prevails in *E. coli* and *K. pneumoniae* strains.^39^^,^^40^ For this reason, fluoroquinolones should be considered only in documented infections caused by quinolone-susceptible ESBL-PE.^41^ In addition, ESBL-PE can develop coresistance to aminoglycosides mainly through aminoglycoside-modifying enzymes coproduced by CTX-M ESBL on the same plasmid.^42^ In our study, coresistance to amikacin and gentamycin was diagnosed in (24.5% and 44.3% of cases) and to ofloxacin in 88.7%. However, cross-resistance to piperacillin/tazobactam was found in 38.7% of cases. This suggests the possibility of piperacillin/tazobactam use in case of complicated UTI caused by ESBL-PE. Indeed, Seo et al.^43^ found a clinical and microbiological response to piperacillin/tazobactam treatment in 94% of cases with UTI caused by ESBL producing *E. coli*, similar to the response to ertapenem treatment. Chastain et al.^8^ found that renally eliminated antibiotics can achieve sufficient urinary concentrations for effective eradication of organisms determined to be resistant per in vitro susceptibility testing. This led some authors to rethink antibiotic treatment strategies for UTIs in the era of antimicrobial resistance.^44^^,^^45^

Our study has two significant limitations. First, it deals with the resistance profile of the isolated microorganisms independently of the clinical diagnosis of the UTI and the prior exposure to antibiotics. Second, it is monocentric and was focused on the capital city of French Guiana and its surroundings (the center and the east of the department) and deserves to be widen toward the other population basins, notably the west of French Guiana, which is more subject to cross-border exchanges. However, it shows the local microbial ecology, which should be considered in daily practice when selecting empirical treatment in case of community-acquired UTI.

## CONCLUSION

Our study shows that the most isolated microorganisms from outpatient urinalyses were *Enterobacterales*, mainly *E. coli* and *K. pneumoniae*. They showed a high resistance rate to amoxicillin but susceptibility to most remaining antibiotics. Additionally, our study shows a decreasing susceptibility profile in *Enterobacterales* for amoxicillin/clavulanic acid, cefotaxime, and nitrofurantoin across the 16 quarters of the study. Interestingly, nitrofurantoin, and fosfomycin were active against most GNRs and can be considered as empirical treatment of uncomplicated UTIs. Fluoroquinolones are active against most GNRs and *Enterobacterales* isolates, reflecting a low antibiotic pressure in the community in French Guiana. However, they should be spared in the first- and second-line UTI treatment. This study is helpful for clinicians to guide the empiric treatment of outpatients with UTI symptoms in our region. Moreover, it would help local authorities in developing antibiotic policies for treating UTIs. Further clinical investigations are needed to identify predictive factors and outcomes of patients with community-acquired UTI in the Amazonian region.
